# A Next-Generation Human Lymphatic Filariasis Vaccine Candidate, r*Bm*HAXT, for Clinical Development

**DOI:** 10.21203/rs.3.rs-6572437/v1

**Published:** 2025-06-21

**Authors:** Nithila Saravanan, Sean Gray, Jennifer Davis, Conrad M Puff-Carter, Vishal Khatri, Nikhil Chauhan, Darrick Carter, Ramaswamy Kalyanasundaram

**Affiliations:** University of Illinois at Rockford; PAI Life Sciences, Inc; PAI Life Sciences, Inc; PAI Life Sciences, Inc; University of Illinois at Rockford; University of Illinois at Rockford; PAI Life Sciences, Inc; University of Illinois at Rockford

**Keywords:** Neglected Tropical Disease, Lymphatic filariasis, vaccine, rBmHAXT, cysteinyl residue mutations, stability study, immunogenicity, vaccine-induced protection, TLR-4 agonist

## Abstract

This study was conducted to yield a robust and scalable manufacturing process for a candidate vaccine for human lymphatic filariasis (LF) - a tropical parasitic infection transmitted by mosquitoes. In previous studies, we demonstrated that removing an affinity purification tag from the fusion protein did not affect immunogenicity or vaccine efficacy. During scaled-up production of r*Bm*HAXT, we noticed that significant amounts of the antigen aggregated, resulting in the loss of purified vaccine antigens. Thus, this project aimed to create new r*Bm*HAXT forms more suitable for industrial-scale production while maintaining robust protection. We generated three different variants: one with all the cysteinyl residues mutated to serinyl residues (delta-Cys), a second one with a flexible glycine-serine linker inserted between each of the component antigens (GS), and finally, a third variant with a combination of both the cysteine deletion and the addition of linkers (delta-Cys GS). We then evaluated the immunogenicity and efficacy of each variant in a mouse model. We demonstrated that the delta-Cys mutant retained immunogenicity and vaccine efficacy similar to the parent tag-free r*Bm*HAXT protein. We also evaluated the proteins in an accelerated stability study at five (5) different temperatures (−80°C, −20°C, 4°C, 25°C, and 40°C). We concluded that all preparations were stable at 4°C, and the delta-Cys variant was stable even at 25°C up to the completion of the study (6 weeks). In addition to improved stability, the delta-Cys protein exhibited reduced aggregation and equivalent potency in mice and, therefore, is an optimal candidate for progression to cGMP manufacturing and human clinical trials as a vaccine for lymphatic filariasis.

## INTRODUCTION

Lymphatic filariasis (LF) is a mosquito-transmitted tropical parasitic infection affecting over 81 million people in 51 countries worldwide, with an additional 882 million people in 44 countries at risk of acquiring the disease^1-3^. There are no vaccines available to control this infection. Preventative chemotherapy consists of three medications currently administered through a mass drug administration (MDA) is the current approach to control this infection in endemic regions^4^. This MDA approach significantly reduced the disease burden in a few countries,^1^ but has not eliminated the disease, despite 24 years of this approach. Only partial disease elimination has been achieved partly due to patient non-compliance, shorter half-life of the drugs used in MDA, little or no effect on the adult worms, and reemergence of the infection in several endemic regions^5-8^. An effective vaccine complementing MDA would benefit significantly, especially since a distribution infrastructure has been established for MDA^9^.

In previous studies, we demonstrated the efficacy of a tetravalent recombinant fusion protein vaccine, which resulted in 85% protection in mice and 57% protection in non-human primates^9-11^. Since these worms do not multiply within the human host, a substantial decrease in worm establishment can significantly reduce infection-associated pathology, as was shown in the monkey trials^11^. Additionally, lower worm burdens and decreased fecundity of female worms following the immunization can result in fewer microfilariae in the circulation^12^ and thereby will function as a transmission-blocking vaccine that synergizes with MDA. Given the promising results in the pre-clinical studies, we initiated scale-up production for the tag-free recombinant vaccine antigen for industrial manufacturing under cGMPs^13^. During this process, we found significant aggregation of the proteins, especially following purification and upon storage at refrigerated conditions (2–8°C). To improve the recovery of less aggregated recombinant protein, we generated three different variants of the antigen in this report. We then tested each variant for their vaccine potency, stability, immunogenicity, and efficacy compared to the parent vaccine candidate. Our results showed that one variant, where all cysteinyl residues were mutated to serinyl residues, had minimal to no aggregation, better stability, and proved to be a better vaccine candidate in terms of its potency.

## RESULTS

### Expression levels of rBmHAXT variants

**Figure 1:**
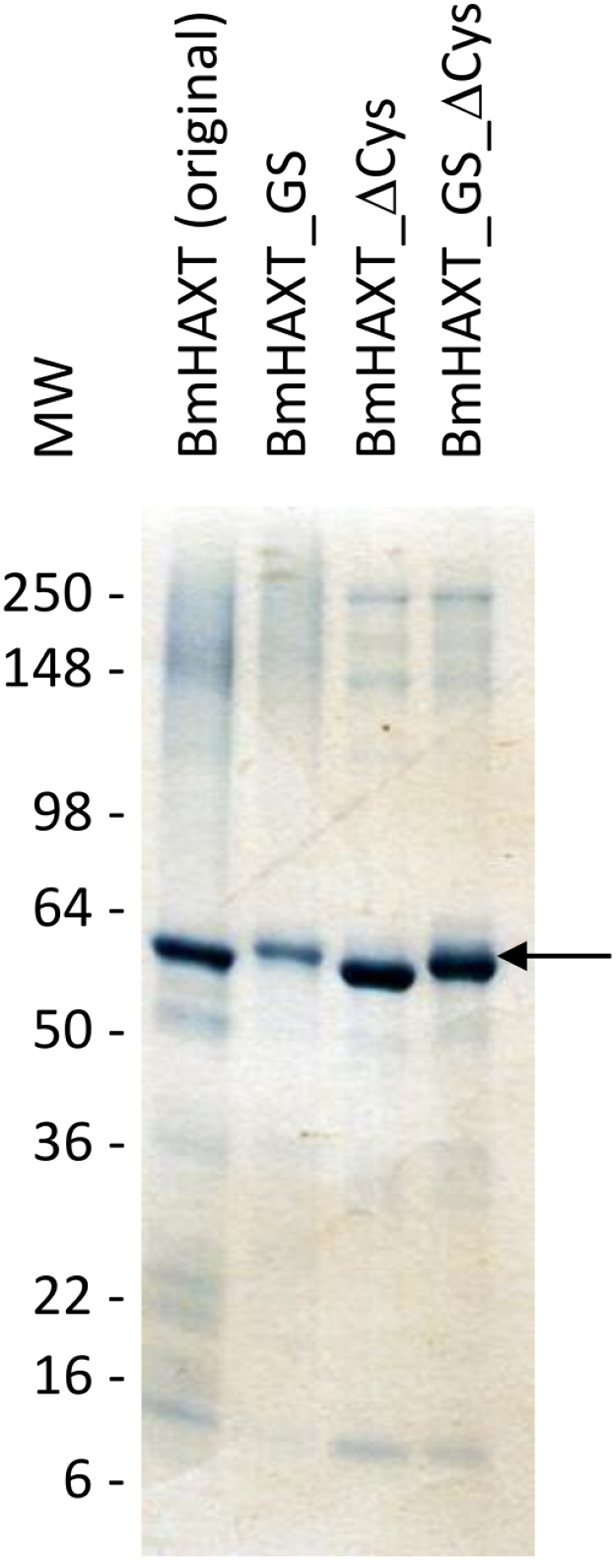
Expression and purity of rBmHAXT mutants. Approximately 2.5 μg of each of the r*Bm*HAXT variants, r*Bm*HAXT original (Lane 1), r*Bm*HAXT (GS) (Lane 2), r*Bm*HAXT (ΔCys) (Lane 3) and r*Bm*HAXT (ΔCys+GS) (Lane 4) were separated on a Novex^™^ Wedge Well^™^ 4-20% Tris-glycine gel and stained with Simply Blue^™^ Safe stain. SeeBlue PLUS2 molecular weight markers were used to determine the molecular mass. There was a major band at 60 kDa in all samples. However, the original protein also had several bands around 140 - 250 kDa (possibly aggregates) and several fragments around 10- 25 kDa. Comparatively, there were fewer aggregates and fragments in all other mutants, with greater than 97% purity for the 60 kDA bands for r*Bm*HAXT (ΔCys) and r*Bm*HAXT (ΔCys+GS) mutants.

With respect to purity of the four proteins, we found that the cysteine-deleted variants were significantly purer with respect to the primary band at 65 kDa (Fig. 1). The r*Bm*HAXT (ΔCys) and r*Bm*HAXT (ΔCys + GS) also seemed to be significantly improved - particularly with respect to degradation bands in the 16–50 kDa region. The original protein exhibited the greatest amount of initial aggregation and the greatest number of bands below 65 kDa. Expression levels of the variants containing the glycine-serine linkers were consistently lower than levels observed with r*Bm*HAXT (ΔCys).

### ΔCys variant of rBmHAXT is more stable

**Figure 2:**
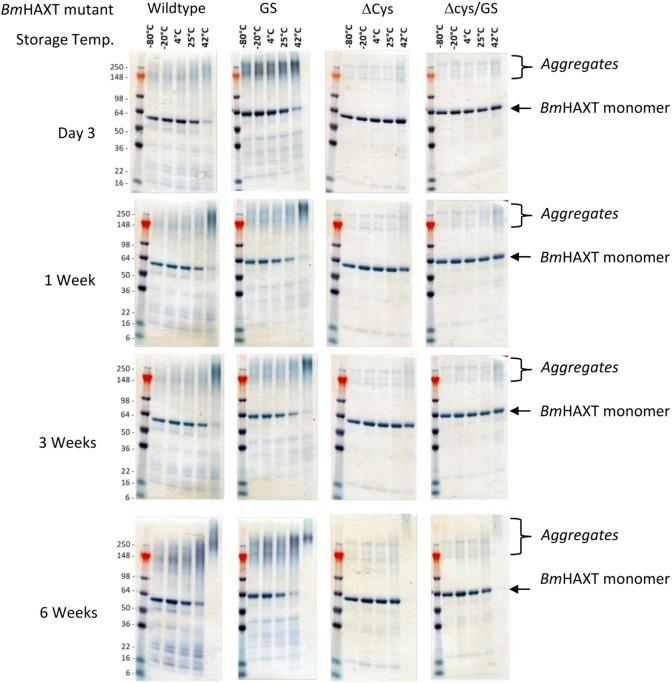
Stability of r*Bm*HAXT mutants at various temperatures. Single use aliquots of each of the r*Bm*HAXT mutants, r*Bm*HAXT (original), r*Bm*HAXT (GS), r*Bm*HAXT (ΔCys), and r*Bm*HAXT (ΔCys+GS) were stored at −80°C, −20°C , 4°C , 25°C and 42°C for 6 weeks (completion of the experiment). Approximately 2.5 μg of the samples were removed on day 3, week 1, week 2, week 3, week 4 and week 6 and were separated on a 4-20% Tris-glycine gel and stained with Simply Blue^™^ Safe stain. SeeBlue PLUS2 molecular weight markers were used to determine the molecular mass. Our data show that the r*Bm*HAXT (original) has a propensity to aggregate with major aggregate band around 150 - 200 kDa. At 2 weeks, the r*Bm*HAXT (original) and r*Bm*HAXT (GS), were nearly completely aggregated at 42°C, whereas, more than half of r*Bm*HAXT (ΔCys) and r*Bm*HAXT (ΔCys+GS) remained intact at 25°C. Based on these observations, we conclude that the removal of cysteine residues greatly reduced aggregation of r*Bm*HAXT protein and improved the overall stability (with and without the GS linker).

We performed accelerated stability studies at five (5) temperatures for 6 weeks. For brevity and ease of comparing profiles between time points, the Day 3, 1 week, 3 weeks, and 6 weeks, time points are only shown in Fig. 2 with the monomeric band and aggregates highlighted. After one week, the original r*Bm*HAXT as well as the r*Bm*HAXT (GS) were nearly completely aggregated at 42°C, whereas, more than half of r*Bm*HAXT (ΔCys) remained as monomers. At three weeks, both r*Bm*HAXT and r*Bm*HAXT (ΔCys) were fully aggregated at 42°C. At six weeks, over 90% of the r*Bm*HAXT (ΔCys) protein remained intact at 25°C. Addition of a GS linker did not have the intended effect, which was to promote protein folding and decrease aggregation. Instead, it seemed to have an opposite effect and resulted in more aggregation, which was observable after 3 days and more pronounced at the 25°C and 42°C temperatures (Fig. 2). Moreover, the aggregates also seemed to be slightly larger (~ 300–400 kDa) than what was observed for r*Bm*HAXT (ΔCys). Based on these observations, we conclude that the removal of cysteine residues greatly reduced aggregation of r*Bm*HAXT protein and improved the overall stability (with and without the GS linker).

### All the variants of rBmHAXT induced significant antibody titers

**Figure 3:**
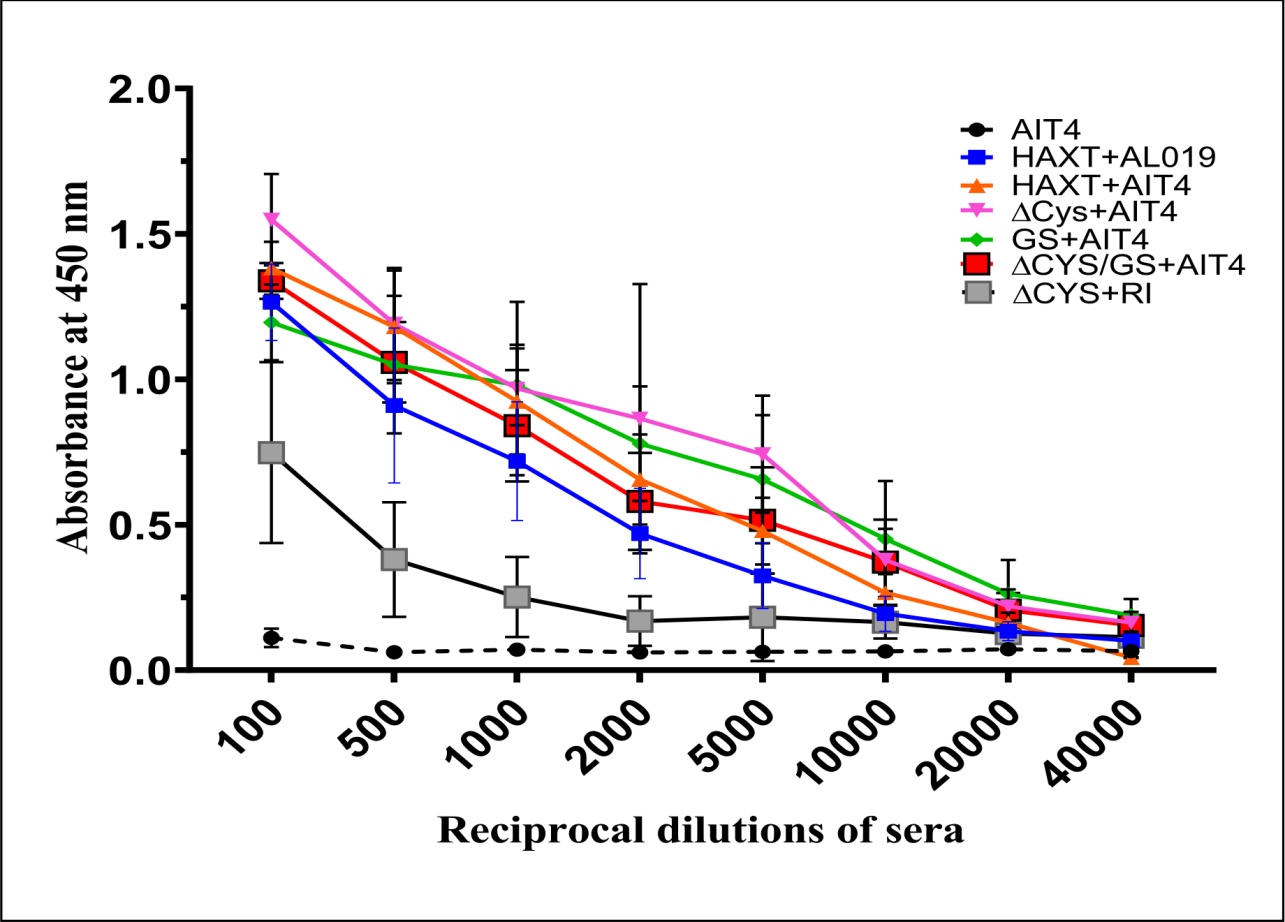
Titers of antigen-specific IgG levels after three immunizations with r*Bm*HAXT variants. Titers of antigen-specific IgG were evaluated in the sera samples of mice immunized three times with the respective antigens using a capture ELISA. Samples were analyzed on duplicate wells. Our results show that the titers of antigen-specific IgG antibodies were significantly increased in all vaccinated groups (***p<0.0001) compared to the adjuvant control group (1:20,000). The r*Bm*HAXT (ΔCys) group had a slight but significant increase (#p<0.01) in IgG levels compared to the tag-free protein group. Statistical significance between groups was determined by two-way ANOVA with Tukey’s multiple comparisons test. Each data point represents Mean± SD value. N=5 mice per group.

The results showed that compared to control mice that were given adjuvant alone, all mice vaccinated with r*Bm*HAXT (tag-free) or r*Bm*HAXT (ΔCys) or r*Bm*HAXT (GS), or r*Bm*HAXT (ΔCys + GS) showed significantly (p < 0.005) higher titers of r*Bm*HAXT-specific IgG antibodies compared to controls in the sera of mice (Fig. 3) confirming that ΔCys or GS or the combined modification did not significantly alter the immunogenicity of the r*Bm*HAXT vaccine antigen. These studies also suggested that the immune epitopes of the r*Bm*HAXT vaccine antigen were not altered despite the cysteine mutation or addition of GS linker sequence.

At 1:5,000 dilutions, titers of IgG were significantly (p < 0.05) higher in the sera of r*Bm*HAXT (ΔCys) immunized mice compared to r*Bm*HAXT (tag-free) immunized mice. IgG antibodies were detectable even at 1:20,000 dilutions of the serum samples in both r*Bm*HAXT (tag-free) and r*Bm*HAXT (ΔCys) immunized mice. In our ELISAs, we coated the wells with the parent his-tagged r*Bm*HAXT antigen. Significant binding of the r*Bm*HAXT (ΔCys)-specific IgG to the parent his-tagged r*Bm*HAXT suggests that the r*Bm*HAXT (ΔCys) retains the immunogenic epitopes of the parent antigen. These findings confirm that redesigned r*Bm*HAXT (ΔCys) retains immunogenicity relative to wild-type protein.

**Figure 4:**
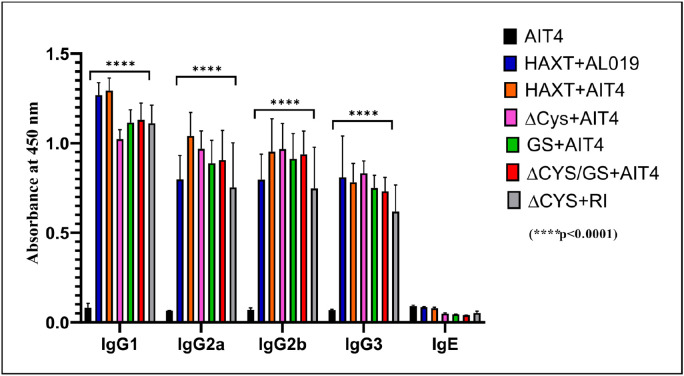
Levels of antigen-specific IgG antibody isotypes and IgE antibodies in immunized mice sera. Antibody isotype profiling of the vaccinated mice revealed that the levels of all IgG antibody isotypes were significantly elevated (****p<0.0001) across all groups when compared to the adjuvant control group. No significant differences were observed in the levels of antibody isotypes between the immunized groups. The IgE levels were same as background readings in all immunized mice. Statistical significance was determined by two-way ANOVA with Tukey’s multiple comparison test. Each bar represents Mean ± SD value. N=5 mice per group.

Antibody isotype analysis also showed that the levels of IgG1, IgG2a, IgG2b, and IgG3 isotypes were elevated in the sera of all immunized mice (Fig. 4) and were highly significant (****p < 0.0001). However, the IgE antibody levels were at background levels, demonstrating that none of the variants induced an IgE antibody response in mice. In summary, these studies showed that immunization with the variant proteins did not affect immunogenicity with respect to total IgG or IgG subclasses.

### Antigen-specific splenocytes from vaccinated animals secreted high levels of IL-17A and IFN-γ

**Figure 5:**
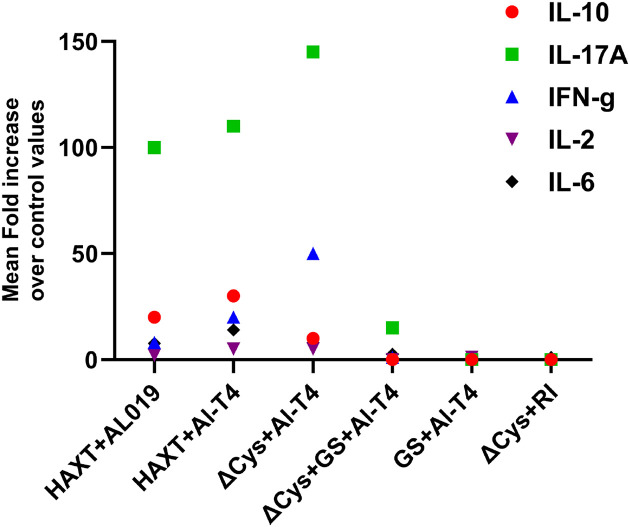
Secreted levels of cytokines in the culture supernatants of r*Bm*HAXT-specific splenocytes. Spleen cells collected from immunized mice were stimulated with 5 μg of rBmHAXT for 72 hrs, and culture supernatants were assayed in a flow cytometer using a cytokine bead array kit from BD BioSciences. Values shown are fold increases over the control values from mice that received only the adjuvant. The secreted levels of IL-17A, IL-2, IL-10, and IFN-γ in r*Bm*HAXT+AL019 and rBmHAXT +AlT4^™^ groups showed similar patterns of increases. However, the cells from r*Bm*HAXT (ΔCys)+AlT4^™^ immunized animals secreted nearly 150 fold higher levels of IL-17A and about 50 fold higher levels of IFNγ suggesting that a potent Th17/Th1 response is induced by r*Bm*HAXT (ΔCys)+AlT4^™^ immunization. Statistical significance was determined by two-way ANOVA with Tukey’s multiple comparisons test. Each bar represents Mean ± SD value. N=5 mice per group.

Cytokines level in the culture supernatants of spleen cells were determined using a cytokine bead array. Our results showed that while comparing r*Bm*HAXT + AL019 and r*Bm*HAXT + AlT4^™^, a similar pattern of cytokine response (IL-17A, IFN-γ, IL-10) was observed (Fig. 5).

However, compared to AL019 adjuvant, the AlT4^™^ adjuvant appears to boost the levels of these secreted cytokines potentially suggesting that AlT4^™^ may have a slightly better adjuvanting effect on r*Bm*HAXT compared to AL019. When the cytokine response to r*Bm*HAXT + AlT4^™^ was compared with r*Bm*HAXT (ΔCys) + AlT4^™^, IL-17A and IFN-γ response was substantially higher in r*Bm*HAXT (ΔCys) + AlT4^™^ compared to r*Bm*HAXT + AlT4^™^ suggesting that the ΔCys mutant promoted better Th17/Th1 responses following vaccination, consistent with the lower IgG1 levels seen with this combination (Figs. 4 and 5). The IL-10 response was slightly lower, but several fold higher than the controls. Spleen cells from the other mutants secreted very low levels of cytokines tested. These findings show that AlT4^™^ is a more efficient adjuvant for r*Bm*HAXT and in combination with r*Bm*HAXT (ΔCys), AlT4^™^ promoted a robust cellular responses (Figure S2).

#### T _CM_ cells were generated in the spleen of mice vaccinated with rBmHAXT or its ΔCys mutant protein.

**Figure 6:**
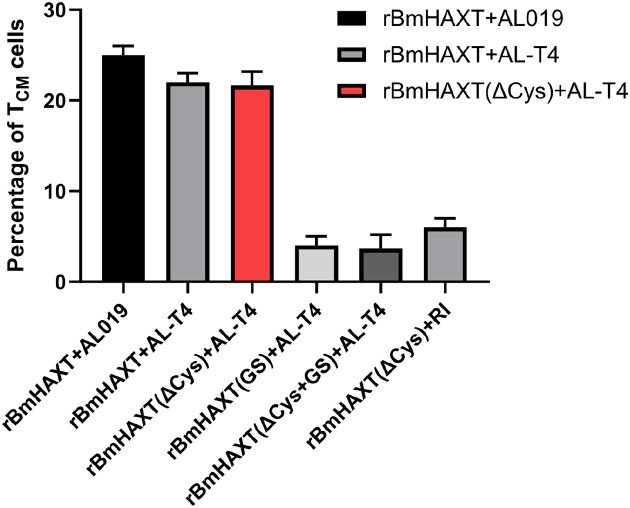
Percentage increase in r*Bm*HAXT-specific T central memory (T_CM_) cells in the spleen of immunized animals. Animals immunized with the parent protein or ΔCys protein showed significantly higher increases in the percent of r*Bm*HAXT-specific T_CM_ cell population in their spleens. However, animals immunized with r*Bm*HAXT(GS), r*Bm*HAXT(ΔCys+GS) or r*Bm*HAXT(ΔCys)+RI group had only very small increases in the r*Bm*HAXT-specific T_CM_ cells in their spleens. Each bar represents Mean ± SD value. N=5 mice per group.

To evaluate if the vaccination generated memory T cells, we cultured 1 × 10^6^ splenocytes from each mouse with 5ĝ/ml of r*Bm*HAXT protein for 72 h at 37°C. Central memory T cells express CD62L and CCR7 receptors. CD62L or L-selectin is a marker that distinguishes central memory (Tcm, CD62L+) from effector memory (Tem, CD62L^−^) T cells. C-C receptor 7 (CCR7) is a chemokine receptor that plays a role in the homing of central memory T cells to the spleen and lymph nodes. CCR7 is absent in effector memory T cells. The central memory cells circulate through the lymphatic system and have the ability to self-renewal and provides significant immunity against pathogens. After incubation, cells were harvested and stained with CD3/CD62L/CCR7 antibodies and analyzed in a flow cytometer. After gating the cells for T (CD3^+^) cells, cells that were dual positive for CD62L and CCR7 were counted as T_CM_ cells. Our results showed that mice vaccinated with r*Bm*HAXT or r*Bm*HAXT (ΔCys) generated a significant percentage of T_CM_ cells irrespective of the adjuvants (AL019 or AlT4^™^) used (p ≤ 0.0001) (Fig. 6).

### Vaccine-induced protection

**Figure 7:**
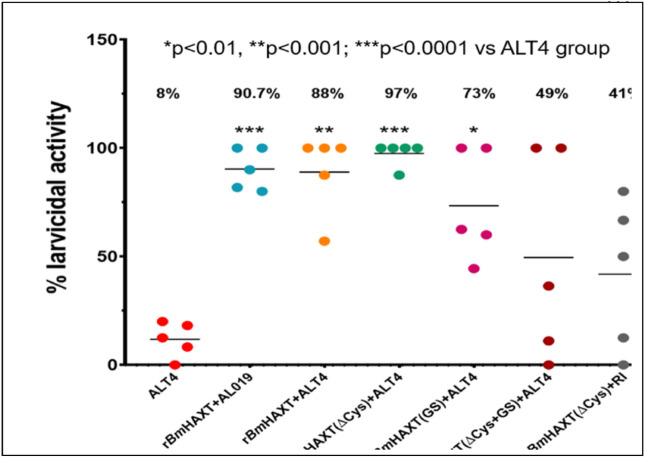
Immunization with r*Bm*HAXT and its variants conferred significant levels of protection in vaccinated animals. Vaccine-induced protection was determined by calculating the percentage of larval death in immunized animals. Following three immunizations, the percent protection in r*Bm*HAXT(Cys)+ALT4^™^ and r*Bm*HAXT+AL019 was significantly higher (***p<0.001) compared to the adjuvant group. The vaccine-induced protection was also significantly (*p<0.1) higher in the r*Bm*HAXT(GS) group and the r*Bm*HAXT(ΔCys+GS) group compared to the control group. However, the level of protection in these groups was significantly lower compared to the r*Bm*HAXT(ΔCys) and r*Bm*HAXT vaccinated animals. Statistical significance was determined by one-way ANOVA. N=5 mice per group.

We then evaluated the protective potential of the vaccine antigens using a challenge model. Challenge studies in mice showed that the percent protection was comparable or even higher in mice immunized with r*Bm*HAXT (ΔCys) compared to the parent r*Bm*HAXT vaccine antigen (Fig. 7). Mice immunized with r*Bm*HAXT or r*Bm*HAXT (ΔCys) showed 97% protection compared to adjuvant control (8%), r*Bm*HAXT (GS) group (73%), r*Bm*HAXT (ΔCys + GS) group (49%) and r*Bm*HAXT (RI) group (41%). These findings suggested that the r*Bm*HAXT (ΔCys) retained the vaccine efficacy of the parent vaccine (r*Bm*HAXT) antigen. This study also showed that the cysteine mutation did not affect the vaccine efficacy of r*Bm*HAXT.

## DISCUSSION

Developing a r*Bm*HAXT-based vaccine for human clinical trials required that we remove the 6 × his tag used during the research production process. However, the tag-free r*Bm*HAXT showed significant aggregation and numerous lower molecular weight fragments following purification. To address this, we generated three different mutant mutants of r*Bm*HAXT termed r*Bm*HAXT ΔCys, r*Bm*HAXT GS, and r*Bm*HAXT ΔCys + GS. With the ΔCys variant, we tested our hypothesis that r*Bm*HAXT by virtue of its non-natural fusion of 4 protein domains, has a propensity to aggregate likely due to adjacent cysteinyl residues forming disulfide bonds between separate protein chains rather than the native disulfide bonds required for proper folding. We therefore replaced all cysteinyl residues with serinyl residues. Our second hypothesis was that direct fusion of the four proteins in the wildtype protein hampers the freedom and space for each of the four subunits to properly fold, and this misfolded nature would promote aggregation. Therefore, GS variant proteins containing a 15 amino acid flexible glycine-serine linker were also produced to allow the chains to fold independently of each other. We produced each of the four proteins and analyzed them for expression levels, stability, and resistance to aggregation using a five temperature 6-week accelerated stability program. We tested the potency, immunogenicity, and vaccine efficacy of each variant to select the best r*Bm*HAXT candidate for industrial manufacturing and clinical studies. Our results show that out of the three variants tested, r*Bm*HAXT (ΔCys) appears superior to the parental (his-tagged or tag-free) r*Bm*HAXT protein in its immunogenicity and vaccine efficacy in a mouse model.

Recombinant proteins, especially cysteine-rich proteins, tend to form protein aggregates leading to the formation of inclusion bodies (IBs) during translation in *E. coli*^14,15^. Formation of IBs can be both problematic and beneficial when it comes to scale up and manufacture of a parasite recombinant protein ^14, 17-18^. One problem is that the IBs must be solubilized in urea and then refolded *ex vivo* to form active protein monomers should an enzymatically active protein be desired. However, IBs are heavy compared to solutes in lysed bacteria, and a significant benefit to IB formation is that once the bacteria are lysed the IB fraction can be purified separately from the soluble fraction leading to initially semi-pure recombinant protein. In the context of r*Bm*HAXT, our process takes advantage of, and in fact promotes the formation of IBs. The goals of our study were to produce high purity antigen, that primarily forms stable monomers, will maintain a non-aggregated state for extended periods of time, and will promote a robust, protective vaccine response. We are not looking for enzymatic activity, nor are we looking for properly folded soluble protein, both due to the fact that r*Bm*HAXT is a synthetically engineered non-native protein made up of 4 diverse domains. Wildtype r*Bm*HAXT tended to aggregate significantly which we hypothesize was due to the 17 cysteinyl residues forming non-natural disulfide bonds that is cysteines in one subdomain forming a disulfide bond with cysteines in a second domain. We also hypothesized that the non-natural nature of the protein made it difficult to fold *in vivo* in *E. coli*, especially while large amounts are being translated during IPTG induction. Slight misfolding during this process may result in aggregation. Thus, in this project, removal of all cysteine residues helped reduce the possibility of forming protein aggregation. However, contrary to our hypothesis, introduction of flexible glycine serine linkers did not promote better folding and instead led to increased aggregation and decreased protein yields. This was in contrast to our hypothesis that by increasing the distance between the component proteins, the subdomains would have the space and freedom to fold independent of each other which would lead to decreased aggregation and increased expression in the soluble fraction. Similarly, the combination of the GS linkers and the cysteine mutations was also not as beneficial as the cysteine modification alone. We demonstrate that we were able to purify recombinant monomer protein up to > 95% pure. Thus, these findings clearly suggested the advantage of mutating all cysteine residues for preventing protein aggregation and allowing more efficient purification.

The vaccine is intended for subjects living in the tropical countries where LF infection is highly prevalent. Thus, the vaccine protein needs to remain stable in events of interrupted cold storage and preferably at higher temperatures during transportation and storage. Vaccines can unstable during storage and this instability can reduce the safety and efficacy of the vaccines being deployed in certain areas ^17,18^. The Expert Committee for Biological Standardization provides guidance on thermal stability and shelf life of vaccines during manufacturing ^19, 20^. Based on these guidelines and in an effort to move the vaccine to clinical use, we tested the stability and shelf life of our recombinant vaccine antigens in this study. Our results show that the r*Bm*HAXT ΔCys protein is stable - even at 25 °C, when tested at 6 weeks, the completion of our accelerated stability study, whereas, all other formulations started aggregating and fragmenting within 2 weeks at 25 °C. These data suggest that the improved vaccine is more stable at various temperatures, which is advantageous during transportation and storage.

Following any improvement to the vaccine antigen there is a need to test the formulation to determine if the vaccine retains the immunogenicity and vaccine efficacy of the original formulation ^21, 22^. The mouse is a good model to evaluate the immunogenicity and efficacy of vaccine candidates against lymphatic filariasis ^23-26^. Thus, when we tested the immunogenicity of all three mutants in a mouse model, we found that all three mutants are highly immunogenic. The r*Bm*HAXT ΔCys elicited a robust IgG response comparable to the parent r*Bm*HAXT protein and the type of IgG isotype responses were also similar to that of the parent r*Bm*HAXT molecule, confirming that the new mutants are equally immunogenic as the original tag-free formulation. Our studies also demonstrated that the IgG antibodies generated against each of the mutants cross-react with the parent his-tagged r*Bm*HAXT suggesting that the key immunogenic epitopes are not altered with the mutations or sequence alterations. This implied that we could use any of the variants as a vaccine candidate to replace the parent formulation.

It is critical to determine if any or all of the mutants can confer a protective immune responses against lymphatic filariasis. The preclinical studies reported here confirmed that all the mutants are able to generate protective immune responses at varying levels. However, the protective immune responses generated following vaccination with r*Bm*HAXT ΔCys was higher compared to the rest of the mutants and even the parent tag-free r*Bm*HAXT. This suggested that the cysteine mutations had no influence on the immunoprotective epitopes of r*Bm*HAXT. This also suggested that the cysteinyl residues are not critical for vaccine-induced immunity or that the serinyl residues - due to their chemical similarity - are suitable mimics of the native cysteine structures. Analysis of splenocytes from immunized mice show that all of the immunized mice generated T central memory cells. Thus, deletion of cysteine residues in r*Bm*HAXT did not interfere with the generation of memory T cells in vaccinated animals.

Several previous studies show that both antibodies and cellular responses are critical for host immunity against lymphatic filariasis ^26-32^. Our previous studies showed that when infective larvae of *B. malayi* were incubated with immune sera and PBMC or peritoneal cells from mouse ^10^, gerbil ^12^, rhesus macaque ^11,33^ or humans ^34^, plenty of macrophages and lymphocytes bound to the larval surface that led to the death of the larvae within 24–48 hrs ^9^. If cells alone, immune serum alone, or IgG depleted immune human serum were used, this killing does not happen ^34^. This suggests that both IgG and cells (mainly macrophages) are critical for the killing of the larvae ^9^. Results from our ADCC assay in this study also show significant larval death when incubated with the immune sera. Sera from mice immunized with r*Bm*HAXT (ΔCys) had the highest percent of larval death suggesting that immunization with r*Bm*HAXT (ΔCys) induces protective IgG antibodies and/or effector cell types.

Adjuvants play a critical role in eliciting and skewing the immune responses favorable to maximum protection ^35-37^. Thus, in this study, we also tested three different adjuvant formulation to determine which formulation gives the best protective response. While the TLR4-agonist-on – alum adjuvants are relatively similar and activate inflammatory responses via alum, and also activate TLR4 with the respective agonists, the other one, RI, is known to be a potent combination adjuvant. The two TLR4-on-Alum formulations gave significantly better results than the RI adjuvant. This could be due to the fact that RI induces too much of a response leading to immune downregulation, consistent with the lower responses seen in our assays. The new formulation generated by our group, AlT4^™^ gave the best results suggesting that the new formulation has significant potential as an adjuvant for human vaccines for lymphatic filariasis.

## CONCLUSION

Our studies demonstrated that mutation of cysteine residues in our vaccine protein could significantly improve the quality of the vaccine antigen. Cysteine mutation did not affect the expression of the protein; in fact, it improved the recovery of the purified antigen. Our studies also showed that the ΔCys mutation significantly improved the stability of the vaccine antigen. Similarly, vaccine efficacy studies in a mouse model showed that mutation of all cysteine residues in r*Bm*HAXT ΔCys did not affect the immunogenicity, potency, or vaccine efficacy - if anything these changes boosted responses. Significantly, we also were able to show a next generation alum/TLR4 adjuvant, AlT4^™^, gave the best responses in our test systems. Overall, our results demonstrate that we now have an optimal version of our LFGuard^™^ candidate and are poised for cGMP production and human testing of this important vaccine.

## MATERIALS AND METHODS

### Design and Cloning of rBmHAXT (tag-free)

To ensure traceability from clone development throughout process development, all cloning, expression, and purification reagents were carefully sourced and documented to ensure the absence of animal-derived products used in the process. Tag-free r*Bm*HAXT was prepared as described by Melendez *et al.*

### Design and Cloning of rBmHAXT (ΔCys)

After production of r*Bm*HAXT (tag-free), we found that the protein rapidly forms large aggregates. This led to reduced solubility and stability during large scale purification. To address this issue, a third generation candidate, referred to as r*Bm*HAXT (ΔCys), was designed *in silico* by changing all cysteine codons in the *Bm*HAXT sequence to serine codons to reduce crosslinking by disulfide bonds and oxidation of the cysteinyl residues. There are a total of 17 cysteinyl residues in r*Bm*HAXT interspersed amongst the four component protein sequences. The reasoning behind mutating all cysteine residues was: (1) this will reduce the formation of disulfide bonds that could increase aggregation due - in part - to aberrant disulfide bond formation during the refolding process ^14^; (2) since r*Bm*HAXT is a fusion of four independent protein sequences, the potential to form disulfide bonds between a cysteinyl residue in one protein to a cysteinyl residue in another protein due to close proximity during folding can be avoided ^15^; (3) similarly, aggregation of r*Bm*HAXT may potentially suggest that the individual proteins are not able to achieve their native fold and therefore would reduce potency since antibodies may not recognize the natively folded protein in the parasitic worm; (4) the sulfur atoms in the cysteines can oxidize themselves leading to suboptimal stability; and finally (5) the cysteinyl residues are probably not critical for the generation of a protective immunity since the serinyl residues likely bind to the same MHC molecules in a similar manner and do not interfere with that binding through the formation of interchain disulfides ^16^ For the mutagenesis, cysteine codons with the nucleotide sequence TGC were mutated to AGC serine codons by changing the thymine base in the first position to an adenine base while cysteine codons with the nucleotide sequence TGT were mutated to TCT serine codons by changing the guanine base in the second position to a cytosine base (**Table S1**).

The redesigned r*Bm*HAXT (ΔCys) gene was manufactured as a GeneBlock^™^ gene fragment from Integrated DNA Technologies (IDT, Coralville, IA). The r*Bm*HAXT (ΔCys) was cloned into the pET29a(+) expression vector (Millipore Sigma, Burlington, MA). Plasmids were transformed into *E. coli* Turbo cells (NEB, Ipswich, MA) and the transformants were selected on LB plates supplemented with 50 ug/mL of kanamycin sulfate (LB-Kan). The transformants were screened by PCR for correctly sized inserts and the presumptive positive sequences were confirmed by Sanger sequencing. Plasmid from a single confirmed clone was transformed into the commercial *E. coli* expression strain HMS174 (DE3) and transformants selected on LB-Kan plates. Resulting colonies were screened for expression of a~ 60 kDa band corresponding to the predicted size of r*Bm*HAXT (ΔCys).

### Preparation of Additional Variants of rBmHAXT

Two other variant proteins termed r*Bm*HAXT (GS) and r*Bm*HAXT (ΔCys + GS) were designed as possible next generation candidates. Both r*Bm*HAXT (GS) and r*Bm*HAXT (ΔCys + GS) include three 12 amino acid flexible glycine-serine (GS) linkers (Gly-Gly-Gly-Ser-Gly-Gly-Gly-Ser-Gly-Gly-Gly-Ser) inserted between each of the four protein sequences in the fusion. The intended purpose of the linkers were to promote native folding of each individual protein. In order to prevent internal crossing over of the genes due to the presence of the identical linkers, the nucleotide sequence of the 3 glycine linkers were all intentionally designed differently by randomizing as many of the codons for the 12 amino acids. For r*Bm*HAXT (ΔCys + GS), we mutated all 17 cysteine residues in r*Bm*HAXT (GS) to serine residues (**Table S1)**. The only difference was in the numbering for each cysteine residue as it differed slightly for r*Bm*HAXT (ΔCys + GS) due to the insertion of the glycine-serine linkers. Similar to r*Bm*HAXT (ΔCys), both r*Bm*HAXT (GS) and r*Bm*HAXT (ΔCys + GS) were designed *in silico,* produced as GeneBlocks, and cloned and purified as described below.

### Fermentation

Fermentation was optimized at the two liter scale and a fermentation batch record was developed. Briefly, a cell bank vial was inoculated into 2 × 200 mL of LBK broth and grown to a cell density of >3 OD_600nm_. Protein expression was induced by addition of isopropyl ß-D-1-thiogalactopyranoside (IPTG) to a final concentration of 1 mM. Agitation-induced foaming was minimized by constant addition of Antifoam 204 (Sigma, St. Louis, MO) to a concentration of 0.01% throughout the growth and induction phase. Fermentation was performed at 37±1°C, with air flow at 30 L/min, and pH maintained at 7.0 +/− 0.2 by addition of 6 N NH_4_OH (base) or 5 N HCl (acid) as needed. Dissolved oxygen was held at a minimum of 40% by cascading with agitation followed by oxygen supplementation. Harvest by centrifugation was performed 3 hours post-induction.

### Process Development

The process for purifying r*Bm*HAXT (ΔCys) was same as described by Melendez *et al.*^13^, with two modifications: first, we did not use dithiothreitol in the purification of r*Bm*HAXT (ΔCys) since we had deleted all the cysteine residues in this protein and therefore would not need a reducing agent for disulfides. The second modification was to reduce the washing stringency (NaCl concentration reduced from 180 mM to 160 mM) during the purification washing steps. The reason for this step was that some r*Bm*HAXT ΔCys protein was being washed away and reducing the salt concentration improved final yields without decreasing overall purity.

### Isolation of Inclusion Bodies (IBs)

*E. coli* cell pellets were thawed and resuspended in 5 mL lysis buffer (50 mM tris and 0.5% Triton X-100 pH 8.0) per gram of wet cell paste and mixed by vortexing and disruption by pipetting until no visible clumps were observed. The suspension was passed 3 times through a LM10 microfluidizer (Microfluidics Corp., Westwood, MA) at 15,000 psi allowing for intermittent cooling between passes. The IB fraction was pelleted by centrifugation at 14,000 × *g* for 30 min. The IB pellet was resuspended in 20 mL of 1% 3-[(3-cholamidopropyl) dimethylammonio]-1-propanesulfonate (CHAPS) detergent solution per gram IB and then pelleted by centrifugation as above. The IB pellet was resuspended in 20 mL 25% isopropyl alcohol per gram of IB and pelleted again as above. Washed IB pellet was resuspended in 20 mL of solubilization buffer [50 mM tris, 8 M urea pH 8.0] per gram of IB and rolled gently at 4°C for 16–20 hours. The solubilized crude IB solution was clarified by centrifugation at 15,000 × g for 3 hours at 4°C. The supernatant containing the solubilized r*Bm*HAXT (ΔCys) was decanted to a fresh container and stored at −80°C until purification.

### Purification and Diafiltration of Recombinant Proteins

Capto Q ImpRes ion exchange chromatography was used to purify r*Bm*HAXT (ΔCys). Solubilized IB solution (180 mL) was passed across a strong anion exchange resin, Capto Q ImpRes (Cytiva, Marlborough, MA) at a flow rate of 10 mL min^−1^. The resin was washed to baseline with 4–5 column volumes (CV) of Q Wash Buffer (50 mM tris, 8 M urea, 160 mM NaCl, pH 8.0). Protein was eluted until the UV trace came to baseline using 1–2 CV of Q Elution Buffer (50 mM tris, 8 M urea, 300 mM NaCl pH 8.0). The emerging peak was analyzed by SDS-PAGE to confirm the enrichment of the r*Bm*HAXT (ΔCys) protein. The Capto Q elution fraction - enriched in r*Bm*HAXT (ΔCys) protein - was then loaded onto a Capto SP ImpRes (Cytiva) strong cation exchange resin. The eluted protein pool was diluted 1:8 with SP loading buffer (20 mM acetate, 8 M urea pH 4.0). The adjusted solution was loaded at a flow rate of 4 mL min^−1^. The resin was washed until the UV trace came to baseline with 3–5 CV of SP Wash Buffer (20 mM acetate, 8 M urea, 300 mM NaCl pH 4.0) to remove non-specifically bound contaminant proteins. Protein was eluted with 1.5–2 CV of SP elution buffer (20 mM acetate, 8 M urea, 1 M NaCl pH 4.0). The emerging peak was analyzed by SDS-PAGE to confirm further enrichment of r*Bm*HAXT (ΔCys) protein.

Pooled protein was buffer exchanged into 20 volumes of 50 mM Tris pH 8.0 by tangential flow filtration using Pellicon^®^ 10 kDa MWCO diafiltration cartridges (Millipore Sigma) and then concentrated to 2–4 mg mL^−1^ as measured by OD_280nm_ absorbance. Glycerol was added to 5% (v/v), the final concentration adjusted to 1 mg mL^−1^, and sterile filtered through 0.2 μM filters.

Triplicate 1 μg loads of r*Bm*HAXT (ΔCys) and BSA standards were analyzed by reducing SDS-PAGE and quantified by ImageJ densitometry analysis to confirm both concentration and purity. Identity was confirmed by Western blot analysis with monkey anti-r*Bm*HAXT antisera ^11^ at 1/10,000 and detected with a 1/10,000 dilution of HRP-conjugated Goat anti-Monkey IgG (H + L) secondary antibody (Thermo Fisher Scientific, Rockford, Il.). Presence of residual *E. coli* host cell proteins were detected by Western analysis using a 1/1,000 dilution of Rabbit anti-*E coil* Host Cell Protein (HCP) polyclonal antibody (Rockland Immunochemicals, Inc., Limerick, PA) detected with a 1/2,000 dilution of HRP-conjugated Donkey anti-Rabbit IgG (H + L) secondary antibody (Southern Biotech, Birmingham, AL). Residual endotoxin was measured using the *Limulus* amebocyte lysate (LAL) assay and read using the Endosafe^®^ Nextgen PTS Reader (Charles River Laboratories, Worcester, MA).

### Stability of rBmHAXT Protein and its Variants

A short-term stability study was performed to compare the tag-free r*Bm*HAXT with the three variants: r*Bm*HAXT (ΔCys), r*Bm*HAXT (GS), and r*Bm*HAXT (ΔCys + GS). The stability study was performed by diluting each of the two proteins to 0.5 mg/mL in 20 mM Tris pH 8.0. Approximately 40 aliquots of 0.1 mL of each protein were placed at the five indicated temperatures. Protein stored at −80°C in a Revco Ultra Low freezer was used as a control condition given that there should be no change in aggregation or degradation at this temperature. Storage at −20°C was in a standard freezer. Storage at 4–8°C was used to simulate a typical refrigerator. The 25°C temperature simulates typical room-temperature conditions. Finally, 42°C is chosen to promote forced (accelerated) degradation and was meant to stress test the proteins at a higher temperature. At each of the seven time points, one aliquot of each of the proteins was removed from each of the five storage conditions and 1 μg total protein was resolved on a reducing SDS-PAGE gel and stained with SimplyBlue Safe Stain (Thermo Fisher Scientific).

### Cell Banking

A research cell bank of 162 vials of *E. coli* HMS174 (DE3) strain containing plasmid r*Bm*HAXT ΔCys clone 6B in vector pET29a was laid down. The clone was grown in animal product free LB broth supplemented with 50 ug/mL of kanamycin sulfate (LBK) to an OD600 of ~ 1.0. After confirming the culture purity by microscopic observation and growth on both selective and non-selective media, the culture was mixed 1:1 with sterile LBK broth containing 20% plant-derived glycerol. The cell bank was stored at −80°C. Confirmation of expression before and after cell banking was performed and the expressed protein was confirmed to be localized to the insoluble fraction (IBs) as expected. Using the newly produced research cell bank (RCB), a 200 vial GMP Master Cell Bank (MCB) was produced at the University of Nebraska Biological Process Development Facility (BPDF). We confirmed the expression of r*Bm*HAXT ΔCys protein from the newly created MCB (data not shown).

### Immunogenicity and Vaccine Efficacy of the rBmHAXT variants

Vaccine proteins r*Bm*HAXT, r*Bm*HAXT (ΔCys), r*Bm*HAXT (GS), and r*Bm*HAXT (ΔCys + GS) were expressed as recombinant proteins and purified as described above. The major focus of the animal studies were to determine if mutating all cysteinyl residues in r*Bm*HAXT or adding a glycine-serine linker in between the protein subdomains would result in increased or decreased immunogenicity *in vivo* compared to the parent protein.

### Adjuvants

In this study, we used three different adjuvants: (i) Aluminum hydroxide combined with GLA (AL019) obtained from the Access to Advanced Health Institute (Seattle, WA); (ii) Aluminum hydroxide combined with a TLR4 agonist (AlT4^™^) prepared by PAI Life Sciences (Seattle, WA); and (iii) a potent adjuvant based on the original RIBI adjuvant system^38^ termed PAI-RI.

The alum-adsorbed TLR4 agonist is produced in a two-step process. First, a micellar suspension of the TLR4 agonist is produced (MiT4^™^) which is also used as a TLR4-only control. This suspension is then adsorbed to the surface of Alhydrogel^™^ by mixing. 3D-(6-acyl) PHAD (Croda/Avanti Polar Lipids, Yorkshire, UK) is added to a flask, then combined with 1,2-dipalmitoyl-sn-glycero-3-phosphocholine (DPPC), and dissolved in chloroform. Chloroform is then evaporated, water for injection is added, and the mixture placed in a sonicating bath at 60°C. Periodically, samples are taken and analyzed by Dynamic Light Scattering using a Malvern Zetasizer^™^. Once a suitable particle size averaging approximately 120 nm is obtained, the MiT4^™^ is sterile filtered and aliquoted. Next, Alhydrogel (Croda, Yorkshire, UK) is washed in water and sedimented by centrifugation at 3500 RPM for 25 minutes at 4°C. MiT4^™^ from the previous step is then added and the mixture vortexed at room temperature for 25 minutes until thoroughly combined. Once combined, the AlT4^™^ adjuvant is aliquoted into single use vials and capped for storage at 2–8°C.

The final adjuvant, PAI-RI, was originally produced as a replacement for RIBI as this adjuvant is no longer commercially available. PAI Life Sciences prepared the custom RIBI-like adjuvant composed of a squalane emulsion containing cell wall skeleton from *Mycobacterium phlei,* monophosphoryl lipid A (MPLA) from *Salmonella minnesota* R595, and trehalose 6,6'-dimycolate (TDM) from *Mycobacterium bovis*^38^. PAI-RI has recently shown significant promise in preclinical studies for a tri-antigen syphilis vaccine ^39^.

### Animals and Parasites

Eight-week-old male BALB/c mice purchased from Taconic biosciences (Hudson, NY, USA) were quarantined for two-weeks before the start of the experiments at the University of Illinois College of Medicine Rockford animal facility. Use of animals in this study was approved by the institutional animal care and use committee (IACUC) of the University of Illinois, Rockford. Euthanasia of animals were performed with carbon dioxide gas followed by cervical dislocation as per the recommendation in the ‘Guide’ following the National Institutes of Health guidelines for the care and use of laboratory animals. The infective larval stage (L3) of *B. malayi*were purchased from the TRS Laboratories (Atlanta, GA).

### Vaccination Protocol

Thirty five (35) mice were divided into seven (7) groups of five mice each. Group 1 mice received 10 μg of the TLR4 on Alum (AlT4^™^) adjuvant in 100 μl of PBS. Group 2 mice received 25 μg of r*Bm*HAXT plus 10 μg of AL019 adjuvant; Group 3 mice received 25 μg of r*Bm*HAXT plus 10 μg of AlT4^™^ adjuvant; Group 4 mice received 25 μg of r*Bm*HAXT (ΔCys) plus 10 μg of AlT4^™^ adjuvant; Group 5 mice received 25 μg of r*Bm*HAXT (GS) plus 10 μg of AlT4^™^ adjuvant; Group 6 mice received 25 μg of r*Bm*HAXT (ΔCys + GS) plus 10 μg of AlT4^™^ adjuvant; and Group 7 mice received 25 μg of r*Bm*HAXT (ΔCys) plus 10 μg of PAI-RI. All injections were given subcutaneously (SC) into the right flank region of each mouse on days 0, 14 and 28.

### Collection of Blood

Approximately 100 μL of whole blood was collected from the submandibular vein of each mouse on day 0 (pre-immune), day 14 (before first booster), day 28 (before second booster) and on day 48 (before challenge). Mice were anesthetized with a ketamine / xylazine formulation (0-100 mg/kg / 5–10 mg/kg) before collecting the blood. Serum samples were prepared and stored at μ80°C for serological analysis.

### Titer of Antigen-Specific IgG

The titer of r*Bm*HAXT-specific IgG in serum samples were evaluated using an indirect ELISA as described previously ^10^. Briefly, wells of a 96 well plate were coated overnight at 4°C with 1 μg/mL of his-tagged r*Bm*HAXT. After washing the plates with phosphate buffered saline containing TWEEN-20 (PBST), the wells were blocked with 3% bovine serum albumin. Following this, diluted (1:100, 1:1,000, 1:5,000, 1:10,000, 1:20,000, and 1:40,000) serum samples were added and incubated for 1 h at room temperature. Following incubation, the plates were washed with PBST, and HRP-conjugated chicken anti-mouse IgG antibody (Thermo Fisher scientific) at 1:10,000 dilution was added as the secondary antibodies. Following 1 hr incubation at room temperature, plates were washed with three rounds of PBST and distilled water before adding 1-step Ultra TMB-ELISA substrate and 0.16 M H_2_SO_4_, (Thermo Fisher Scientific) and optical density was determined at 450 nm using a BioTek Synergy 2 ELISA reader.

### Levels of Antigen-Specific Antibody Isotypes

Levels of r*Bm*HAXT-specific antibody isotypes (IgG1, IgG2a, IgG2b, IgG3, IgE, IgM and IgA) were determined in the serum samples using an indirect ELISA as described above ^10^. Respective isotype-specific biotinylated goat anti-mouse antibodies (Sigma) at 1: 10,000 dilution and streptavidin-HRP (1:20,000) was used as secondary antibodies. Color was developed with 1-step Ultra-TMB. The reaction was stopped using 0.16 M H_2_SO_4_ and absorbance was determined at 450 nm using a BioTek Synergy 2 ELISA reader.

### Challenge Studies

To determine vaccine-induced protection, we used a micropore chamber challenge method as described by Chauhan *et al.*^10, 13^. Briefly, 20 infective larvae of *Brugia malayiwere* placed in a micropore chamber and surgically implanted into the peritoneum of each mouse. Seventy two hours following implantation, the chambers were recovered from the mice and the total number of larvae recovered were counted. The larvae were then examined under a phase contrast microscope for adherence of cells and for larval death. Larvae that were transparent, straight, and with no movement were counted as dead. Larvae that were active, coiled, and translucent were counted as live.

### Secreted Levels of Cytokines from Antigen-Stimulated Splenocytes

After removing the chambers, spleens were collected, and single-cell suspensions made from each mouse. Approximately, 2 × 10^6^ cells in duplicate wells were stimulated with 5 μg/ml of r*Bm*HAXT for antigen-specific proliferation. Cells stimulated with 2 μl/ml concanavalin A (ConA) were used as a control for non-specific stimulation. Unstimulated splenocytes were kept as a negative control for the assay. After 72 h of incubation, culture supernatants were collected and levels of IL-2, IL-4, IL-6, IFNγ, TNFα, IL-10, and IL-17A were determined using a cytokine bead array kit (BD Bio Sciences, San Jose, CA).

### T cell Subsets in Antigen-Stimulated Splenocytes

Isolated splenocytes were washed and labeled with fluorescently labeled anti-mouse CD3 (APC) and within the CD3 gated population, the CD62L (PE/Cya7) and CCR7 (PE) positive T cells were identified as T-central memory cells. The percent population of each cell type was determined using a flow cytometer. Briefly, cells were incubated with FcγRII blocker in staining buffer (2% FBS + 0.1% sodium azide) for 30 minutes at 4 °C. Following washing of the cells, all three fluorescent-labeled antibodies were added and incubated for 1h at 4 °C in the dark. After washing, cells were fixed in 4% paraformaldehyde and analyzed using a BD FACSCalibur^™^ (BD Biosciences) flow cytometer.

### Statistical Analyses

GraphPad Prism version 7.0 (GraphPad Software, San Diego, CA) was used to plot all graphs. Statistical analyses were carried out using SPSS version 26.0 (IBM corporation, Armonk, NY). Data was analyzed for normality using Shapiro-Wilk Test, data sets with a *p* value > 0.05 were considered normally distributed. Following this analysis statistical significance of differences between datasets was determined using suitable parametric or non-parametric statistical tests. A probability (*p* value) of < 0.05 was considered statistically significant.

## Data Availability

All datasets presented in this study are included in the article.
